# Cell-Based Relative Potency of a Respiratory Syncytial Virus mRNA Vaccine Correlates with In Vivo Immunogenicity

**DOI:** 10.3390/vaccines13030326

**Published:** 2025-03-19

**Authors:** Katrina Feller, Hesham Nawar, Liping Song, Amanda Abrams, Liang Shang, Ashley Gruber, Tatyana Yun, Hualin Helen Li

**Affiliations:** 1 Analytical Research and Development, Merck & Co., Inc., West Point, PA 19486, USA; 2 Biostatistics and Research Decision Sciences, Merck & Co., Inc., West Point, PA 19486, USA

**Keywords:** mRNA vaccine, potency assay, immunogenicity

## Abstract

**Background/Objectives:** Potency is a critical quality attribute for vaccine development as well as clinical drug product (DP) lot release and stability testing. Animal studies have the potential to offer conclusive insights about the potency of vaccines by demonstrating technical relevance with respect to the hypothesized vaccine mode of action. However, animal studies are expensive, time-consuming, labor intensive, and, most importantly, involve the use of animals. Therefore, alternative in vitro potency assays should be explored. **Methods:** In this study, female BALB/c mice were immunized intramuscularly with various doses of a respiratory syncytial virus (RSV) mRNA vaccine V171 lots at day 0 and day 21. Vaccine-elicited immune responses were determined by ELISA (post-dose 1) and neutralizing assay (post-dose 2). These vaccine lots were also tested in a cell-based relative potency assay in which the ability of each lot to express the RSV F protein in Hep G2 cells was measured against a reference standard. **Results:** Effective Dose 50s (ED50s) of the vaccine lots were determined with probit models based on dichotomized ELISA or neutralizing titers. Statistical analysis demonstrated that the post-dose 2 neutralizing ED50 correlates with cell-based relative potency (Pearson’s correlation test ln (RP) and ln (ED_50_): correlation coefficient = −0.82; *p*-value = 0.047). **Conclusions:** These data merit the use of a cell-based potency assay to replace the animal study to support V171 vaccine development and to use for DP lot release and stability testing. This study also establishes proof-of-concept of using cell-based potency assays as an alternative to animal immunogenicity studies for mRNA-based vaccines.

## 1. Introduction

Human respiratory syncytial virus (RSV) is a leading cause of respiratory illness worldwide. RSV primarily exhibits as lower respiratory tract disease, pneumonia, bronchitis, tracheobronchitis, or upper respiratory tract illness. Anyone can be infected with RSV, but people who are at higher risk for severe illness include infants and young children, everyone over the age of 75 years old, and people over 60 years old who have weakened immune systems, chronic heart and lung disease, or other chronic medical conditions [1].

Recent mRNA vaccines have shown promising results in clinical trials and indeed two mRNA vaccines were approved for emergency use during the COVID-19 pandemic [2,3,4]. Merck & Co., Inc., Rahway, NJ, USA, along with Moderna Inc., have developed a vaccine candidate V171 that encapsulates mRNA encoding RSV fusion (F) protein in lipid nanoparticles (LNPs) [5]. The LNP would deliver the mRNA molecule into the cytoplasm of the cells and prevent them from degradation. Once inside the cells, the mRNA can be translated into RSV F protein to elicit cellular and humoral immune responses. RSV-neutralizing antibody titers and F-binding antibody titers were reported to be inversely correlated with RSV-associated hospitalization [6,7]. Indeed, two RSV F protein-based vaccines have been approved recently to use in adults to prevent RSV infection [8,9].

All biological products must meet prescribed requirements for safety, purity, and potency for regulatory approval. Potency tests, along with a number of other tests, are performed as part of product conformance testing, comparability studies, and stability testing. These tests are used to measure product attributes associated with product quality and manufacturing controls, and are performed to assure identity, purity, strength (potency), and stability of products used during all phases of clinical study. FDA regulations allow for considerable flexibility in determining the appropriate measurement(s) of potency for each product. Potency assays used for release testing of licensed biological drug products must comply with applicable biologics and cGMP regulations. This includes measuring identity and strength (activity) along with potency (biological activity) [10,11,12].

Vaccine development is in scope of this requirement. In vivo testing is preferred over in vitro testing for potency assessment in principle because it is better suited for observing the overall effects of a vaccine on a living subject. Animal studies are used to demonstrate technical relevance with respect to hypothesized vaccine mode of action via total antibody level (ELISA), functional antibody titer (neutralizing), and cellular response (cytokine, T-cell, etc.). However, animal studies are time-consuming, expensive, labor-intensive, and hard to validate. Due to these drawbacks, as well as consideration for the three Rs (Replacement, Reduction, and Refinement), alternative potency assays are encouraged by regulatory agencies.

We previously described the development of a cell-based relative potency assay that was developed to support process development, formulation development, as well as release and stability testing of our RSV mRNA vaccine V171 [13]. The advantage of a cell-based assay in place of an in vivo potency study is the much shorter turnaround time, lower variability, and it helps avoid sacrificing animals. In this study, we tested six lots of V171 drug product in both a mouse immunogenicity study and cell-based relative potency assay to assess the correlation between the mouse immunogenicity and the cell-based relative potency of these lots. The results demonstrate that there is a statistically significant correlation between the cell-based relative potency and the post-dose 2 neutralizing activity in mice. This correlation can be used to support the use of a cell-based potency assay instead of mouse studies for formulation and process decisions, as well as release and stability testing.

## 2. Materials and Methods

### 2.1. Vaccine

V171 was a proprietary mRNA vaccine candidate aiming to prevent RSV infection in the elderly. RSV mRNA encoding RSV F protein DS-Cav1, which is stabilized in its prefusion conformation [14], was produced by Moderna Inc. RSV mRNA was then mixed with four lipids through T Mixing to generate lipid nano particles (LNPs), as described in [15]. The formed LNP drug product intermediate (DPI) was then subject to buffer change, ion exchange chromatography, ultrafiltration, diafiltration, and sterile filtration to generate RSV mRNA vaccine V171 drug product (DP).

### 2.2. Animals

**Figure 1:**
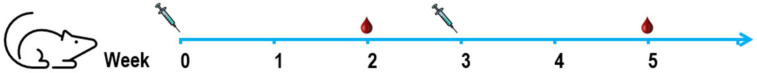
Mouse immunogenicity study design. Six lots of drug product were used to immunize 8 dosing groups of mice. A total of 10 mice were used in each dosing group. Mice were immunized (represented by a syringe) on Day 0 and Day 21, and bled (represented by a blood drop) on Day 14 and Day 35, respectively. The serum from Day 14 bleed was used in the analysis of the ELISA assay and serum from Day 35 bleed was used in the neutralization assay.

Female BALB/c mice (5–6 weeks of age) were purchased from Charles River Laboratories and housed at Covance Research Products Inc., in Denver, PA. This animal study was approved by the Institutional Animal Care and Use Committee (IACUC) at Covance as well as that at Merck & Co., Inc., Rahway, NJ, USA in accordance with animal care guidelines. The mouse study design included eight dosing groups (0.125, 0.25, 0.5, 1, 2, 4, 8, 12 μg mRNA/dose, respectively), each containing 10 mice across six test articles. The mice were immunized intramuscularly with 50 μL per quadricep for a total of 100 μL on Day 0 and Day 21, respectively. Following the immunizations, the mice were observed daily. Test bleeds were performed on Day 14 and Day 35 (Figure 1). The Day 14 sera were collected via retro-orbital bleed and the Day 35 sera were collected through terminal bleed via jugular.

### 2.3. RSV F Protein-Specific ELISA Assay

RSV F-protein LZF60 was prepared as previously described [16] and stored in 50 mM HEPES pH 7.5 buffer supplemented with 300 mM NaCl. The RSV F-protein LZF60 was used to coat 96-well high-binding plates at a concentration of 2 μg/mL in 1 × PBS and incubated at 2–8 °C overnight. On the following day, plates were washed and blocked for 1 h with Li-Cor blocking buffer with 0.2% Tween 20 at room temperature. Serum samples were serially diluted in blocking buffer at 1:90 initial dilution followed by 3-fold serial dilution for eight dilution points. The diluted test articles were then added to the plate at 50 μL per well and incubated at 70–80 rpm for 2 h. Once the 2 h incubation was complete, the plates were washed with PBST (1 × PBS + 0.05% Tween 20). A stabilized peroxidase conjugated goat anti-mouse (Thermo Fisher Scientific, Rockford, IL, USA) was diluted to 0.1 μg/mL in blocking buffer and added to the plate at 50 μL per well and allowed to incubate for 1 h at room temperature. Plates were washed again and developed with SuperBlu Turbo TMB substrate. The reaction was stopped after 6 min with TMB stop solution and plates were read on a SpectraMax M5 (Molecular Devices, San Jose, CA, USA) plate reader at 450 nm.

### 2.4. RSV Neutralization Assay

The RSV-neutralizing assay was conducted utilizing the RSV A2-EGFP (Green Fluorescent Protein-Tagged Respiratory Syncytial Virus) virus which was prepared as described previously [17]. HEP-2 cells were seeded at a density of 20,000 cells/well into a 96-well plate and incubated at 5 ± 1% CO_2_, 37 ± 1 °C overnight. Mouse sera were heat inactivated at 56 °C for 30 min before being diluted 10-fold in medium. Diluted serum was mixed 1:1 with RSV A2-EGFP virus and incubated at 1–1.5 h. After the incubation, the serum-treated virus was added to the HEP-2 cell plates. The infected cells were then placed back into the incubator and allowed to incubate at 5 ± 1% CO_2_, 37 ± 1 °C for approximately 67 h. Plates were then decanted and a 3.7% formaldehyde solution (Polysciences Inc., Warrington, PA, USA) was added to each well. After incubation for 2 min at room temperature, the formaldehyde solution was aspirated using a plate washer and 100 µL of PBS were added to each well. The plates were then read on the SpectraMax i3x MiniMax reader (Molecular Devices, San Jose, CA, USA). The total intensity of the green fluorescent protein expressed in the cells infected with serum-treated virus was used to compare to that of cells infected by RSV A2-EGFP to calculate the percent inhibition for each sample. Samples showing greater than 50% of inhibition of virus infection are determined to be positive for neutralizing activity.

### 2.5. Cell-Based Relative Potency Assay

Cell-based relative potency assay was conducted as described previously [13]. Briefly, test articles and a reference standard were tested at 800 ng/mL mRNA, followed by 2-fold serial dilutions in a 96-well plate pre-seeded with Hep G2 cells. After overnight incubation, cells were fixed with a 3.7% formaldehyde solution before permeabilized with PBS with 1% (*v*/*v*) Triton X-100. The in-house-made RSV F-specific mAb 3D10 was then added to each well at 1 µg/mL in blocking buffer with 0.2% Tween 20 for incubation of 1 h and washed with PBST three times before the addition of 50 µL of IRDye 680RD-conjugated goat anti-human IgG (LICORbio^TM^, Lincoln, NE, USA). The plates were then read on the SpectraMax i3x MiniMax reader (Molecular Devices, San Jose, CA, USA) with transmission light and then with 713 fluorescence light. The data were analyzed with built-in SoftMax (Version 6.5) with 4-parameter logistic regression for percentage of transfected cells and relative potency of the test article was calculated by comparing its EC_50_ to that of the reference standard.

### 2.6. Statistical Analysis

Effective Dose 50s (ED50s) represent the doses of an RSV vaccine lot in which half of the vaccinated mice are considered responders. A responder is defined as a mouse that exhibits sufficient RSV F protein-binding activity in ELISA assay or neutralizing activity in the neutralization assay compared to a naïve mouse. The determination of ED50s of the vaccine lots were conducted using probit models, where the percent of responders served as the response variable, and the natural logarithm of the dose was the explanatory variable. Specifically, an ED50 value was derived by solving the dose level that corresponds to an expected probability of 50% response. The statistical software SAS 9.4 (PROC GLIMMIX) was employed to perform these calculations.

The correlation of the relative potency determined from the cell-based assays and ED50s determined from the probit model using dichotomized ELISA/neutralizing activity of the sample lots was evaluated with Pearson correlation coefficient (*r*). This coefficient measures the strength and direction of a linear relationship between the two sets of measurements. An *r*-value greater than 0.5 typically indicates a strong positive correlation, signifying that as one variable increases, the other variable also tends to increase. If the *r*-value is negative, it signifies a negative correlation, that is as one variable increases, the other variable tends to decrease. If the *p*-value is less than 0.05, it indicates a statistically significant correlation between the two variables under consideration. The statistical analysis was conducted using the R 4.4.0 software program.

## 3. Results

In this study, we assessed the potency of six RSV mRNA vaccine V171 drug product lots in a mouse study for their ability to induce RSV F-specific immune responses. The mouse study design included eight dosing groups, each containing 10 mice across six test articles. The mice were immunized on Day 0 as well as Day 21 and test bleeds were performed on Day 14 and Day 35 for assessment of RSV F-specific ELISA titers and RSV-neutralizing activity, respectively (Figure 1). In the meanwhile, we also tested these samples in a cell-based relative potency assay to assess the ability of the drug product to enter mammalian cells and translate into RSV F protein. We demonstrated the correlation between the cell-based relative potency with the post-dose 2 neutralizing activity in mouse immunogenicity study.

### 3.1. Cut-Off Value Determination for ELISA Titers

A cut-off value was established using data from 30 naïve mouse serum samples across six runs for the ELISA assay. Two analysts ran three separate experiments for a total of six runs to determine an NCO (negative cut-off) value. All 30 samples were prepared at a 10-fold, 20-fold, 40-fold, and 80-fold dilution. Additionally, a positive control generated by pooled sera from 200 mice immunized intramuscularly and a negative control of reagent buffer were run on each plate. Absorbance was read at a wavelength of 450 nm. The NCO value was calculated by taking the average of all samples tested across the six runs and adding it to 1.645 times the standard deviation of all samples. This practice was implemented using the recommendations for the validation of immunoassays used for detection of host antibodies against biotechnology products [18,19].

### 3.2. Total Anti-RSV F-Protein LZF60 Antibodies Titer

**Figure 2:**
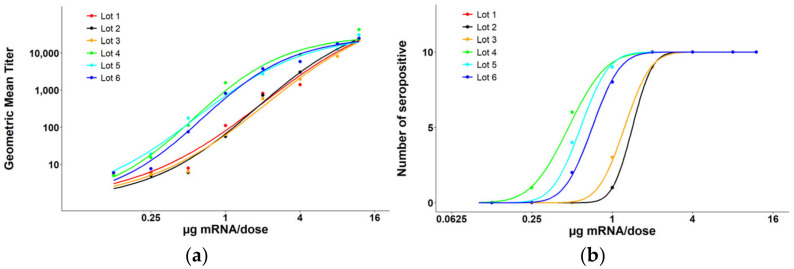
Post-dose (PD1) RSV F-protein-specific ELISA results from the mouse immunogenicity study. PD1 sera were assessed for their ability to bind to RSV F protein LZF60. (**a**) Using endpoint titers for each dosing group, a maximum response was not achieved for all groups and an ED50 value could not be calculated due to a lack of an upper asymptote. (**b**) The number of seropositive responders for each dosing group were used to achieve a full response curve (Lot 1 and Lot 2 completely overlapped). An ED50 value was calculated for each lot.

The mouse serum from the Day 14 bleed was analyzed via ELISA to determine an ED50 value for each test article. A 3-fold dilution of the serum was prepared with eight dilutions and absorbance was read. Endpoint titers were calculated for each mouse and a geomean titer was calculated for all 10 mice in each dosing group. At this timepoint maximum response was not achieved for all test articles (Figure 2a), so an ED50 value could not be calculated based on endpoint titers for each sample. Alternatively, the number of seropositive responders was calculated for each dosing group to determine a full response curve based on the NCO (negative cut-off) value (Figure 2b). An ED50 value (µg mRµNA per dose) as well as a 95% confidence interval (CI) were calculated for each test article (Table 1).

### 3.3. RSV-Neutralizing Antibodies Titers

**Figure 3:**
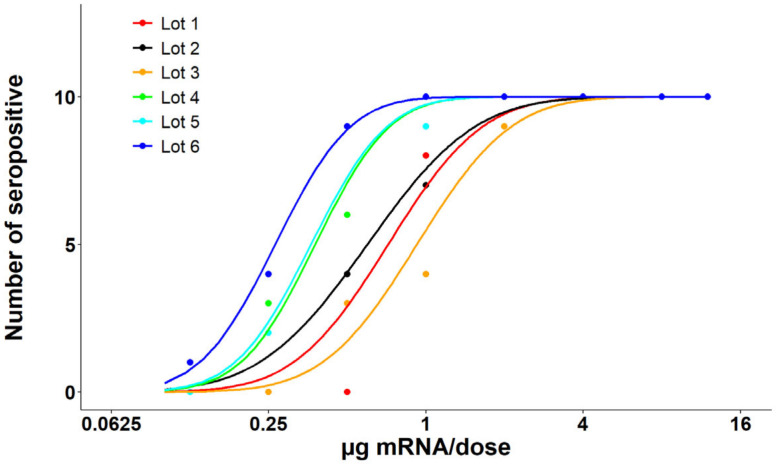
Post-dose 2 (PD2) neutralization assay results from the mouse immunogenicity study. PD2 serum neutralizing activity to RSV virus was determined by RSV A2-EGFP neutralizing assay. Total intensity of the green fluorescent protein expressed in the HEP-2 cells infected with serum-treated virus was used to compare with that from cells infected by RSV A2-EGFP virus to calculate the percent inhibition for each sample. A value over 50% inhibition demonstrated by the serum sample to the RSV virus infection was determined as a positive responder. Full response curves and ED50 values (µg mRNA per dose) are achieved for all lots.

RSV-neutralizing activities of mouse sera from the Day 35 test bleed were analyzed via the neutralization assay. All mouse serum samples were diluted 1:10 and tested in RSV A2-EGFP neutralization assay. The ability of mouse sera to inhibit RSV A2-EGFP virus infection was calculated using fluorescence reading. Total intensity of the green fluorescent protein expressed in the cells infected with serum-treated virus was used to compare with that from cells infected by RSV A2-EGFP virus to calculate the percent inhibition for each sample. A value over 50% inhibition demonstrated by the serum sample to the RSV virus infection was determined as a positive responder. Neutralizing activities were calculated by determining the number of positive responders for each dosing group (Figure 3). An ED50 value (µg mRNA per dose) was calculated for each test article along with a 95% CI (Table 2).

### 3.4. Cell-Based Relative Potency

The six V171 drug product lots were also tested in a cell-based relative potency assay, as described, to assess the ability of the DP to enter mammalian cells and express the RSV F protein [13]. Test articles and a reference standard were tested with serial dilutions in a 96-well plate pre-seeded with Hep G2 cells. Cells were incubated for 16–18 h after transfection and then permeabilized and stained with a human monoclonal antibody specific to RSV F protein, followed by a fluorophore-conjugated secondary antibody. The plate was then analyzed for percentage of transfected cells and relative potency of the test article was calculated by comparing its EC_50_ to that of the reference standard. To minimize variability, samples were tested in four independent runs with duplicate plates for each run. The potency (geomean of four runs) of these six DP lots relative to a reference standard lot ranged from 54.9% to 106.8%, with all relative standard deviation (RSD) below 25% (see Table 3).

### 3.5. Correlation Between Cell-Based Potency and Mouse Immunogenicity

We first assessed the correlation between PD1 ELISA ED50 and PD2 Neutralization ED50 values in the mouse immunogenicity study. Using Pearson’s correlation analysis, the correlation coefficient between PD1 ELISA ED50 and PD2 Neutralization ED50 is 0.77 with a *p*-value of 0.07. An *r*-value greater than 0.5 typically indicates a strong positive correlation, signifying that as one variable increases, the other variable also tends to increase. Although the correlation is not statistically significant at the significance level of 0.05, this result shows that the PD1 ELISA data are trending in a correlative direction to the PD2 Neutralization data.

**Figure 4:**
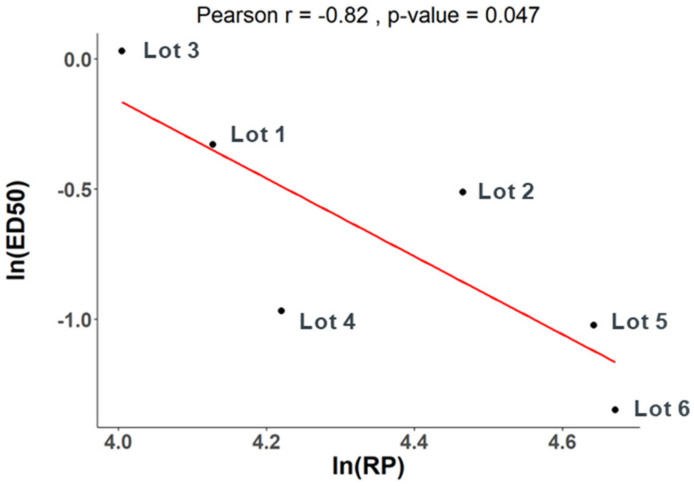
Correlation between cell-based relative potency and PD2 Neutralization ED50. Pearson’s correlation test was used to assess the correlation between cell-based relative potency of six lots of V171 DP and PD2 Neutralization ED50 values from the mouse immunogenicity study. The correlation was determined to be statistically significant with a correlation coefficient of −0.82 with a *p*-value of 0.047.

We then assessed the correlation between the in vivo immunogenicity of these DP lots and their cell-based relative potency (RP) using Pearson’s correlation test ln (RP) and ln (ED50). An *r*-value less than −0.5 typically indicates a strong negative correlation, signifying that as one variable increases, the other variable tends to decrease. In this study, a *p*-value less than 0.05 indicates a statistically significant correlation between the two variables under consideration. The correlation coefficient between PD1 ELISA ED50 and cell-based relative potency is −0.41 with a *p*-value of 0.42. The correlation is not significant at the significance level of 0.05. In contrast, a correlation coefficient of −0.82 with a *p*-value of 0.047 was calculated for the PD2 Neutralization data against the cell-based relative potency. The cell-based relative potency values and the mouse PD2 Neutralization ED50 values are significantly correlated (Figure 4).

## 4. Discussion

Potency is a critical quality attribute for vaccines and therefore needs to be monitored with appropriate method(s). While in vivo studies can reflect the immune responses desired of a vaccine, they require the sacrifice of large numbers of animals and often suffer from long turn-around times, as well as a lack of robustness and reproducibility. Therefore, in vitro potency assays are encouraged to be used when possible. An in vitro potency assay should be able to quantitatively measure the expression of a functionally competent protein antigen, which in turn will lead to in vivo induction of antigen-specific antibodies capable of neutralizing an invading pathogen [20].

In this study, we assessed the in vitro cell-based potency of an RSV mRNA vaccine V171 and the in vivo immunogenicity of this vaccine. We tested six lots of V171 mRNA RSV vaccine drug product in both in vivo mouse study and in vitro cell-based relative potency assay to assess the correlation between the mouse immunogenicity and the cell-based relative potency of these lots. The data obtained in this study demonstrate a clear correlation between the ability of RSV mRNA vaccine V171 DP lots to translate into RSV F protein as measured in the cell-based relative potency assay and in vivo immunogenicity as determined by post-dose 2 neutralizing activity in V171-immunized mice sera. While PD1 ELISA data are trending in a correlative direction to the PD2 Neutralization data, the correlation between cell-based relative potency and post-dose 1 ELISA ED50 values did not reach statistical significance. This could be due to the fact that a lot of animals have not reached the maximum response at this time, and therefore, the number of seropositive responders was calculated for each dosing group to determine a full response and ED50. However, a correlation coefficient (*r*) value of −0.41 does indicate a trend of negative correlation between the PD1 ELISA ED50 and cell-based relative potency.

## 5. Conclusions

In summary, we have demonstrated that the cell-based relative potency assay for our RSV mRNA vaccine V171 is predictive of the ability of the vaccine to elicit a functional immune response in a mouse model. Based on the results of this study, the cell-based potency assay can be considered a suitable replacement for in vivo potency study for development of the V171. This principle can be used for other mRNA vaccines as well.

## Figures and Tables

**Table 1:** RSV F-binding activity (ELISA) seroconversion of animals post-dose 1.

Dose (µg mRNA)	Lot 1	Lot 2	Lot 3	Lot 4	Lot 5	Lot 6
0.125	0	0	0	0	0	0
0.25	0	0	0	1	0	0
0.5	0	0	0	6	4	2
1	1	1	3	9	9	8
2	9	9	9	10	10	10
4	10	10	10	10	10	10
8	10	10	10	10	10	10
12	10	10	10	10	10	10
ED50	1.41	1.41	1.23	0.47	0.58	0.71
95% CI	(1.10, 1.81)	(1.10, 1.81)	(0.93, 1.63)	(0.34, 0.64)	(0.43, 0.77)	(0.53, 0.94)

**Table 2:** Neutralizing activity seropositive animals post-dose 2.

Dose (µg mRNA)	Lot 1	Lot 2	Lot 3	Lot 4	Lot 5	Lot 6
0.125	1	1	0	0	0	1
0.25	0	0	0	3	2	4
0.5	0	4	3	6	9	9
1	8	7	4	10	9	10
2	10	10	9	10	10	10
4	10	10	10	10	10	10
8	10	10	9 *	10	10	10
12	10	10	10	10	10	10
ED50	0.72	0.6	1.03	0.38	0.36	0.26
95% CI	0.52–0.99	0.43–0.83	0.71–1.49	0.29–0.50	0.27–0.48	0.20–0.35

* During centrifugation, a sample tube broke resulting in only 9 samples being submitted for the 8µg dosing group of Lot 3.

**Table 3:** Cell-based relative potency assay results.

Test Article	Cell-based Relative Potency (RP, %)				Geometric Mean RP (%)	ln(RP)	Relative Standard Deviation (%)
	Run 1	Run 2	Run 3	Run 4			
Lot 1	72.5	60.7	58.8	57.1	62.0	4.13	10.8
Lot 2	87.7	71.4	94.5	96.5	86.9	4.47	13.8
Lot 3	67.6	66.2	47.6	42.5	54.9	4.00	23.7
Lot 4	86.1	55.9	67.1	66.3	68.0	4.22	17.9
Lot 5	104.8	116.8	97.8	96.8	103.8	4.64	8.7
Lot 6	115.6	97.1	108.5	106.9	106.8	4.67	7.2

## Data Availability

The datasets presented in this article are not readily available because of the company data management policy. Requests to access the datasets should be directed to the corresponding author.
